# A Framework for Contractual Graphs

**DOI:** 10.3389/fdata.2021.603282

**Published:** 2021-03-04

**Authors:** Renita M. Murimi

**Affiliations:** Gupta College of Business, University of Dallas, Irving, TX, United States

**Keywords:** contractual graphs, Shapley value, kernel, game-theory, smart contracts

## Abstract

This paper studies contractual graphs, where the formation of edges between nodes result in dyadic exchanges. Each dyadic exchange is analyzed as a contractual agreement that is implemented upon fulfilment of underlying conditions. As these dyadic exchanges proliferate, the resulting population of these exchanges creates a contractual graph. A contractual framework for graphs is especially useful in applications where AI-enabled software is employed to create or automate smart contracts between nodes. While some smart contracts may be easily created and executed, others may contain a higher level of ambiguity which may prevent their efficient implementation. Ambiguity in contractual elements is especially difficult to implement, since nodes have to efficiently sense the ambiguity and allocate appropriate amounts of computational resources to the ambiguous contractual task. This paper develops a two-node contractual model of graphs, with varying levels of ambiguity in the contracts and examines its consequences for a market where tasks of differing ambiguity are available to be completed by nodes. The central theme of this paper is that as ambiguity increases, it is difficult for nodes to efficiently commit to the contract since there is an uncertainty in the amount of resources that they have to allocate for completion of the tasks specified in the contract. Thus, while linguistic ambiguity or situational ambiguity might not be cognitively burdensome for humans, it might become expensive for nodes involved in the smart contract. The paper also shows that timing matters—the order in which nodes enter the contract is important as they proceed to sense the ambiguity in a task and then allocate appropriate resources. We propose a game-theoretic formulation to scrutinize how nodes that move first to complete a task are differently impacted than those that move second. We discuss the applications of such a contractual framework for graphs and obtain conditions under which two-node contracts can achieve a successful coalition.

## Introduction

Connections between entities are conveniently represented using graphs. The building blocks of graphs—nodes and edges—have long been used to represent various kinds of networks. The nodes represent entities involved in a transaction, and the edge represents the transaction itself. The interaction between nodes, as denoted by edges, thus represent dyadic exchanges between the nodes. As these dyadic exchanges between nodes in the network increase, it creates a population of these exchanges resulting in a graph. However, any given transaction denoted by a dyadic exchange may not occur until underlying conditions for the implementation of that transaction have been satisfied. In this context, the transaction (represented by a dyadic exchange or an edge in a graph) is not definitive, but is rather an outcome of a set of processes that have to be completed a priori. This paper studies such graphs of such contractual dyadic exchanges and their applications, where the edges between nodes are created only upon fulfilment of underlying clauses. We call such graphs as contractual graphs, since the formation of an edge is similar to the execution of a contract upon fulfilment of underlying conditions.

Contractual graphs are best exemplified by the application of smart contracts, where AI-enabled nodes are tasked with the creation or execution of contracts (Chatterjee et al., 2019). For example, upon transfer of funds from one account to another, the sale of an item is executed or a document is released. In this case, the nodes have to verify if the accounts have sufficient funds, if the transfer has taken place and if the entity has actual control over the item to be sold or the document to be released. Once these have been verified and the operations have taken place, the transaction is completed and can be represented as an edge in the contractual graph (Bartoletti and Pompianu, 2017). For example, the work in (Bigi et al., 2015) describes smart contracts for use in applications such as barter, insurance, escrow, derivatives and general business contracts.

However, not all contracts can be clearly automated. For example, if the contract is executed only upon satisfactory completion of a project, the contract will have to clearly define the threshold for “satisfactory” performance (Schneider, 2019). Any deviation from predetermined values for a task that varies in complexity will require a new smart contract to be created. Examples such as these abound in various domains, where the fuzziness of the contractual language can be parsed by humans, but poses tremendous difficulties while being converted into software. For the aforementioned example of what constitutes *satisfactory* performance, AI-enabled software could help by parsing through large datasets of similar projects that have been labeled as “satisfactory,” “good” or “excellent” and assign the appropriate label. Still, the ambiguity cannot always be resolved using code. Ambiguity, while helping humans achieve leeway in contractual relations, is not a welcome condition in software (Giancaspro, 2017; Clack, 2018). Ordinary qualifiers such as “few,” “some,” or “smart” cannot be programmed effectively without assigning values to these qualifiers. As AI increasingly makes its way into various facets of our lives, it is important to understand that ambiguity in the code can lead to life-altering consequences. For example, when facial recognition software identifies an individual with some probability, the inherent ambiguity in the goal of the task can create adverse outcomes with far-reaching impact (Lohr, 2018). While humans can navigate ambiguity based on situational cues (Eichberger et al., 2009), it is difficult to do so in software (Zou and Zou, 2017). Contractual relationships effected by AI, therefore, have to be cognizant of ambiguity as a key element of the human-machine and machine-machine relationships and a driver of resulting outcomes (Al-Najjar and Weinstein, 2009).

**FIGURE 1:**
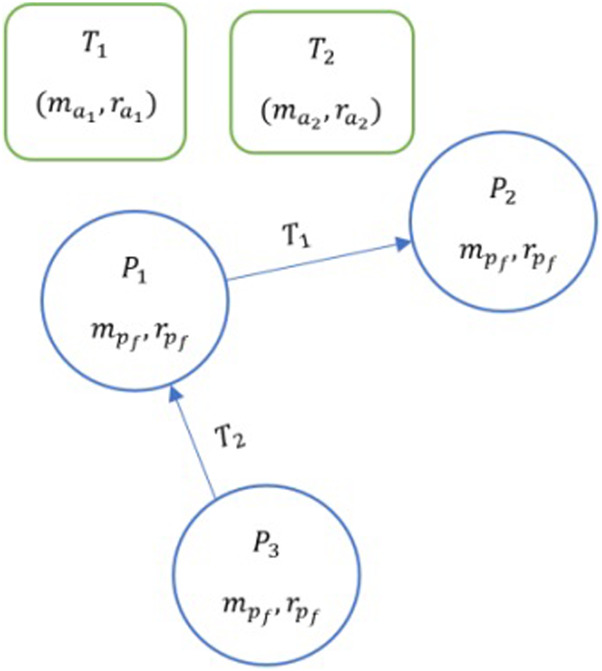
A marketplace with two ambiguous tasks $T_{1}$ and $T_{2}$, each with their levels of ambiguity $\left(m_{a}\right)$ and resource consumption $\;\left(r_{a}\right)$. Three players $P_{1}$, $P_{2}$ and $P_{3}$ assess the tasks to develop their individual perceptions of the ambiguity of a task $\left(m_{p}\right)$ and the allocate resources accordingly $\left(r_{p}\right)$.

In this paper, we study how ambiguity impacts contractual graphs that are represented by dyadic exchanges. Specifically, we use a game-theoretic approach to study how tasks of varying ambiguity are perceived and completed by nodes. To do this, we assume a marketplace with ambiguous tasks (Figure 1). We model the contractual graph as a two-player game, where edges are formed only when two nodes cooperate to complete a task. We show that timing matters, i.e., there are specific advantages to the first mover and the second mover in cooperating to complete the task. Here, two contracts are formed: $P_{3}P_{1}$ commit to task $T_{2}$ and $P_{1}P_{2}$ commit to task $T_{1}$. The direction of the arrow determines the first mover. Here $P_{3}$ is the first mover for task $T_{2}$. $P_{1}$ is the second mover for task $T_{2}$ and is the first mover for task $T_{1}$. Similarly, $P_{2}$ is the second mover for task $T_{1}$. In contract $P_{3}P_{1}$, $P_{3}$ is the first mover and $P_{1}$ is the second mover and are denoted accordingly in the notation.

Next, using the Shapley value, we quantify the value that the first mover and the second mover bring to a coalition. These results are then used to create contractual graphs, whose edges reflect the outcomes of ambiguity and timing in task execution. Further, we provide a representation of such contractual graphs that can be used in learning important information about the community of nodes and the nature of the contracts that are formed in the marketplace.

The rest of this paper is organized as follows. Section *Related Work in Contract Theory and Coalition Value* presents an overview of related work in contract theory and the application of the Shapley value in diverse settings. Section *Model for Contractual Graphs* presents our model for contractual graphs, and Section *Derivation of the Shapley Value for 2-Player Contracts* derives the Shapley value for 2-player coalitions in contractual graphs. Section *Results* presents our findings of simulations. A kernel representation of contractual graphs is presented in Section *Kernel Representation for Contractual Graphs*. Finally, Sections *Future Work* and *Conclusion* present directions for future work and conclude the paper, respectively.

## Related Work in Contract Theory and Coalition Value

The use of game theory to study the mechanism of contract formation has been studied in (Katz, 1990). Here, the author explored contract formation by addressing two questions. First, what are the actions or intentions that are required for a contractual obligation? Second, how do these actions or intentions affect the content of the contract itself? At the heart of this analysis, lies the issue of ambiguity in determining the set of actions or intentions that signal the formation and content of a contract. While legal rules provide a robust framework for determining the outcomes of contracts, the author emphasized the role of social norms and ethical precepts in the outcomes. To this end, the author identified different types of costs involved in contract formation and contract execution and proposes a game-theoretic economic analysis of the steps involved in contract offer, acceptance and rejection.

Another case for the incompleteness of contracts from an economics viewpoint has been made in (Tirole, 1999). Here, the author explains that most contracts are in fact, incomplete. Citing an example of mission and vision statements at higher levels of administration in various domains, the author explains how vague statements such as “increase security” or “provide robust frameworks” are often used to issue directives. The vagueness is inevitable especially since the costs of specifying every contingency and breach can be prohibitively expensive, even if such specifications can be explicitly specified. Such incomplete contract models are characterized by three types of costs: unforeseen contingencies, costs of writing contracts and costs of enforcing contracts. This theme has been continued in (Sklaroff, 2017), where the specific case of inflexibility in smart contracts was studied. The author presented the three features of smart contracts—automation, decentralization and anonymity—that require the formation of fully specified terms for entities to verify the terms of the contract without ambiguity. The author argued that contractual language offers two important attributes—linguistic ambiguity and enforcement discretion that provide powerful efficiencies in the contracting process. This is because smart contracts create transaction costs that are often inflexible. Smart contracts require future stages and terms to be clearly defined beforehand, which is difficult in volatile or unknown environments. Further, generic smart contracts lead to unpredictable and expensive litigation, and create challenging outcomes in cases of breach. Further work in the challenges of smart contracts and the law has been studied in (Verstraete, 2018) where the author presents challenges concerning enforcement and governance in smart contracts, which explicitly eschew central entities in transactions and their outcomes. Another example of developing contracts with predetermined outcomes is in (Asgaonkar and Krishnamachari, 2019) where the authors describe double-sided payment functions in smart contracts to ensure trustworthy transactions.

The aspect of cognitive burden has been addressed in (Tirole, 2015) where the author studies scenarios where having additional information is costly and therefore, players choose scenarios with less information. The paper describes such scenarios as cognitive traps, where the additional cognitive burden imposed by choosing options with higher information is the less desirable option for players. Thus, a player is hurt by choosing the cognitively burdensome option that requires the player to process larger amount of information. The paper further considers cognition-intensive contracting, where the parties to a contract attempt to understand the likely implications of the contract.

The value that players bring to a coalition can be measured in several ways, one of which is the Shapley value. In (Chalkiadakis et al., 2012), the authors present an overview of the applications of the Shapley value in cooperative game theory for AI and computer science. The Shapley value has been used extensively in feature selection in machine learning. Originally derived as a game-theoretic mechanism to characterize the value of each player in a coalition, the derivation of the Shapley value is contingent upon the fulfilment of the following conditions: symmetry (a player’s contribution and not the label assigned to the player is the factor that determines the player’s Shapley value), linearity (utility functions are linear), and carrier (dummy players are assigned a value of zero, and similarly players who make a contribution receive a value that divides the worth of the coalition among players.

The versatility of the Shapley value to diverse applications has led to development in multiple interpretations of the Shapley value. In Sundararajan and Najmi (2019), the authors study several Shapley values for application to a dataset with ten features, and show that the Shapley value with Conditional Expectations was the most sensitive for their application. In Cohen et al., (2005), the authors propose a Contribution-Selection Algorithm (CSA) that ranks each feature according to its contribution value. This algorithm is able to iteratively select n top features with highest contribution values, and is able to remove features with lowest contribution values. Another way in which the Shapley value has been studied is in Bilbao et al., (2008) for the case of bicooperative games. In addition to applications in game-theoretic coalitions, the Shapley value has been used in determining centrality, i.e., the set of most influential nodes in a network (Michalak et al., 2013). Here, the authors describe the application of Shapley value in determining the group of nodes that have the largest influence on the network. Additional work on the role of the Shapley value in social networks is in Narayanam et al., (2014) with applications to community detection and information spread. Other interpretations of the Shapley value include the computation of a bounded rationality Shapley value that ensures that the share of each agent reflects its contribution to the difficulty of computing the coalition values.

While the Shapley value offers a mechanism for determining the value of every player to a coalition, egalitarianism still remains another solution for determining the value of a player. In egalitarian solutions, the worth of each player is determined by equal division. This has led to research that combines the equal division of egalitarian solution vs. the marginalism offered by Shapley value solutions. Work in van den Brink et al., (2013) offers such a concept in the form of egalitarian Shapley values in both cooperative and non-cooperative scenarios.

Coordination games, which refer to the game-theoretic construct of rewarding players when they agree on a common strategy has been extensively studied. A thorough review of the first-mover position in marketing literature is in Kerin et al., (1992). In this paper, the authors described four categories of factors (economic, preemption, technological and behavioral) that contribute to the cost and differentiation advantages of the first-mover position. Examples include partnerships, alliance, or choice of a product. In Jackson and Watts (2002), the authors studied a coordination game in a social network where individuals periodically have the opportunity to add or sever a link.

Literature on coordination in games is extensive (Weidenholzer, 2010), and follows two broad categories. Tacit coordination, in which players communicate only by playing the game, differs from explicit coordination, in which players can send signals that are not directly related to the game (and which may not be costly). The role of communication in facilitation coordination has been studied in Ellingsen and Östling (2010), where the authors showed that communication has the potential to both hamper or hinder coordination efforts.

Work in Apt et al., (2017) studies the specific case of coordination games on finite undirected graphs, which is formulated as a problem where a player has to pick a color from a set of colors. The payoff of the player is the number of other players who pick the same color. Coordination games, here, thus refer to the scenario where it is beneficial for players to align their choices with others in the graph. Another study of a coordination game on a graph is in Rahn and Schäfer (2015), where the authors study the problem of a player having individual preference and may benefit in varying degrees by cooperating with neighbors. Efficient equilibria conditions for extreme cases are considered which allow for deviations, where on the one hand, all players choose the same strategy and on the other hand, each player chooses a unique strategy.

Another study that investigates a population of agents that can dynamically form and sever links according to varying payoffs is in Tomassini and Pestelacci (2010). The resulting co-evolution of strategies results in the formation of connected components in the network. An example of a coordination problem is the Stag Hunt problem, where a hunter must choose to hunt a stag with a group or hunt rabbits by himself (Bryant, 1994). A pure coordination game is described in Van Huyck et al., (1990), where the authors consider a tacit coordination game where strategic uncertainty contributed to inefficient outcomes. A different type of coordination game has been studied in Cason et al., (2012), where group of players are competing against each other. The effectiveness of a group is only as good as that of its weakest player. Thus, this work studies if communication within a group can help or hinder efficiency. Further investigation of strategic uncertainty is in Crawford (1995), in which players repeatedly play coordination games resulting in payoffs that depended on the player’s choices and the summative choices of the other players. A specific group of coordination games is the anti-coordination game (Bramoullé et al., 2004), in which players choose a strategy which is unlike those of her partner. They showed that as the cost of link formation increases, the equilibrium network becomes sparse.

## Model for Contractual Graphs

We now introduce the model to study how ambiguity affects contract modeling by nodes/players in a marketplace. Assume two players $P_{1}$ and $P_{2}$, who both seek a high reputation and are equally abled. The first mover assesses the ambiguity in the contract. Assume that once a player enters a contract, she will complete the task. That is, the model does not study instances where nodes back off after entering the contract. Consequently, the first mover in the contract always commits. We assume a two-player coalition for task completion. Thus, although the first mover has modeled the task in the form of a contract, she requires the help of another player to complete the task. This other player, whom we label as the second mover, has the option to accept/reject offer of coalition with the first mover. For this paper, we study only the case where second mover accepts.

**FIGURE 2:**
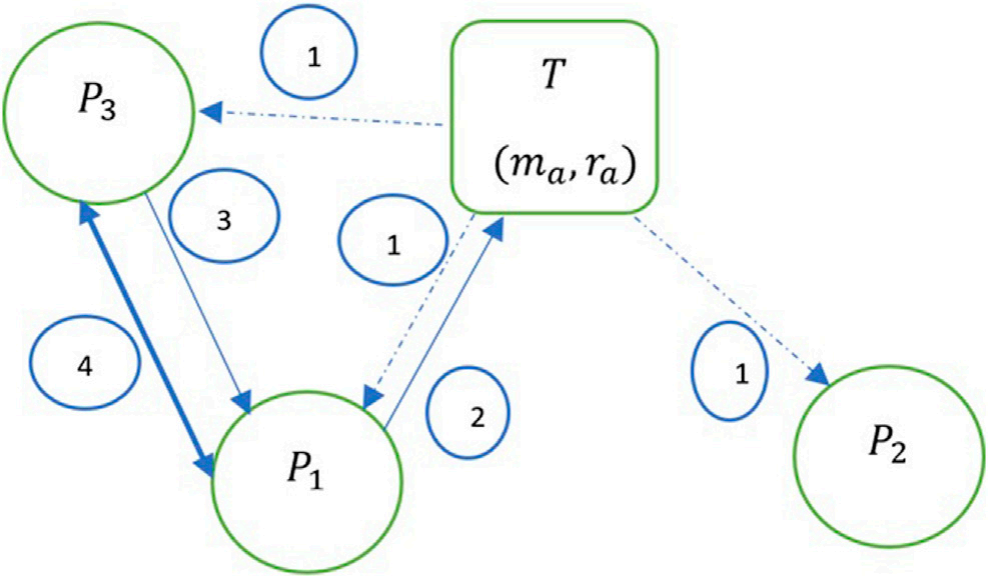
Steps leading to a contract. Assume that a task T appears in the marketplace, with its ambiguity and resource consumption requirements denoted by $m_{a}$ and $r_{a}$ respectively.

Figure 2 shows the steps in the process leading to a contract in a marketplace with one ambiguous task T  and three players $P_{1},\;P_{2}$, and $P_{3}$. The ambiguity and resource consumption of T are given by $\left(m_{a},\;r_{a}\right)$ in order.

Step 1: Players $P_{1}$, $P_{2}$, and $P_{3}$ assess the ambiguity of task T. Each player develops her individual perception of the ambiguity of task T as $m_{p_{i}}$, and accordingly allocates resources $r_{p_{i}}$ for task completion, where i ∈ {1,2,3}.

Step 2: $P_{1}$ decides to create a contract and thereby commits to task T. Since we assume two-player coalitions, $P_{1}$ needs another player to commit to the contract to complete T. Information about this contract is made available to the marketplace as a hard information signal similar to the work in Rajan et al., (2010).

Step 3: The remaining players in the marketplace $\left(P_{2}\;\text{and}\;P_{3}\right)$ receive the signal. Of these two players, $P_{3}$ decides to collaborate with $P_{1}$.

Step 4: The contract is finalized with $P_{1}$ as the first mover and $P_{3}$ as the second mover in the contract.

We assume that players are rational, intelligent and have common knowledge. A player assesses a task’s ambiguity and determines its payoff as the difference between its perception of the work it might be required to do for the task and the actual work that is required for the task. Thus, the initial payoff awareness (IPA) for a node is given by:

Initial payoff awareness (IPA)=Perceived work−Actual work (1)

We define the amount of work to be performed as a product of the ambiguity and the resource commitment. Let the perceived ambiguity for the first mover node be $m_{p_{f}}$. The first mover accordingly allocates $r_{p_{f}}$ resources for the task. Let the actual ambiguity of the task be $m_{a}$, and the resources required for the task be $r_{a}$. Thus, the initial payoff awareness for a first-mover node is given by:

$$IPA_{f}=m_{p_{f}}r_{p_{f}}-m_{a}r_{a}$$ (2)

Similarly, let the perceived ambiguity for the second mover node be $m_{p_{s}}$. The second mover accordingly allocates $r_{p_{s}}$ resources for the task. Thus, the initial payoff awareness for a second mover node is given by:

$$IPA_{s}=m_{p_{s}}r_{p_{s}}-m_{a}r_{a}$$ (3)

We are interested in the difference between the perception of ambiguity and the actual ambiguity. We denote the difference in the perception and actual ambiguity for the first mover as $m_{p_{f}}-m_{a}=\lambda$. Similarly, we denote the difference in the perception of ambiguity and the actual ambiguity for the second mover as $\;\;m_{p_{s}}-m_{a}=\mu$. Further, we assume that the difference in perception of resource consumption for both players is the same, i.e., $r_{p_{f}}-r_{a}=\delta =\;r_{p_{s}}-r_{a}$. Without loss of generality, as we show in the next section of our paper, the constant δ is transformed into a player-specific values $\delta_{1}$ and $\delta_{2}$ for the two players. This transformation drives the derivation of player-specific payoff calculations, and ultimately the derivation of the Shapley value-based representation of the contractual graphs.

We now develop a matrix for the initial payoff awareness for the first mover (Table 1) and second mover (Table 2) based on the difference in ambiguity and resource consumption perception and reality. The table contains four options corresponding to the possible difference in perceptions of ambiguity (high/low), the actual levels of ambiguity (high/low), and the difference in perceptions and actual values of resource commitment (high/low).

**TABLE 1:** Specific conditions enumerating constraints on the initial payoff awareness (*IPA*) matrix for first mover.

Perceived	Actual	μ	δ
High	Low	>0	>0
High	High	=0	=0
Low	Low	=0	=0
Low	High	<0	<0

**TABLE 2:** Specific conditions enumerating constraints on the initial payoff awareness (*IPA*) matrix for second mover.

Perceived	Actual	λ	δ
High	Low	>0	>0
High	High	=0	=0
Low	Low	=0	=0
Low	High	<0	<0

In Tables 1, 2, we assume that each of the four cases are equally probable. This assumption can be revised to dynamically reveal different market conditions, where certain scenarios could be more probable than others. However, for simplicity, this model uses the equiprobable condition for each of these scenarios. Thus, the initial payoff awareness calculations can be obtained from Eqs. 1, 2 and Tables 1, 2 as follows. For the first mover,

$$IPA_{f}=m_{p_{f}}r_{p_{f}}-m_{a}r_{a}$$

Since we assume that a node allocates resources in accordance with the ambiguity, it accordingly allocates higher resources for the task. Similarly, if the node senses lower ambiguity, it allocates fewer resources for the task. This correspondence between the perception of ambiguity and the related resource allocation by a node is shown in Tables 1, 2. For both the first mover and the second mover, if the node senses a high ambiguity, it assigns more resources to the task. For example, in Table 1 for the first mover, the first row denotes that the node senses a high ambiguity, resulting in high difference between the perceived and actual ambiguity denoted by λ. Consequently, it allocates higher amount of resources to the task, denoted by δ. The last row in Table 1 for the first mover corresponds to the case where the node senses a low ambiguity, and accordingly allocates fewer resources to the task (δ). Thus, since the difference in real ambiguity and the perceived ambiguity (λ) impacts the difference in actual and perceived resource consumption, Tables 1, 2 shows cases where both λ and δ are either greater than zero, equal to zero or less than zero, but does not feature opposite cases such as (λ<0  and δ>0 ), or (λ>0  and δ<0 ). A similar assumption is used for the second mover in Table 2.

The first term in the equation below corresponds to the first row for the first mover in Tables 1, 2, where $m_{p_{f}}=m_{a}+\lambda$ and $r_{p_{f}}=r_{a}+\delta$. Thus, the first row indicates that the perceived ambiguity is greater than the actual ambiguity, which is denoted by λ>0 and δ>0 and corresponds to the conditions where $m_{p_{f}}>m_{a}$ and ${r_{p}}_{f}>r_{a}$ respectively. Moving down the table, the second and third rows denote the case where ${m_{p}}_{f}=$ $m_{a}$ and ${r_{p}}_{f}=r_{a}$, thus denoting that λ=0 and δ=0 respectively. These two rows indicate that the perceived and actual ambiguity are equal. Finally, the last row for the first mover in Tables 1, 2 corresponds to the case where perceived ambiguity is less than the actual ambiguity. Thus, λ<0 and δ<0 and corresponds to the conditions where $m_{p_{f}}<m_{a}$ and $r_{p_{f}}<r_{a}$ respectively.

$$\begin{array}{l} IPA_{f}=\frac{1}{4}\left[\left(m_{a}+\lambda \right)\left(r_{a}+\delta \right)-m_{a}r_{a}\right]+\frac{1}{4}\left(0\right)+\frac{1}{4}\left(0\right)+\frac{1}{4}\left[\left(m_{a}-\lambda \right)\left(r_{a}-\delta \right)-m_{a}r_{a}\right]\\ IPA_{f}=\frac{\delta \lambda }{2} \end{array}$$ (4)

Similarly, IPA calculations for the second mover are given by Eqs. 1, 3 and Table 2 as

$$IPA_{s}=\frac{\delta \mu }{2}$$ (5)

Next, we assume that nodes are motivated by reputation, which in turn is a proportional to the number of tasks completed. Reputational incentives or motivations have been extensively studied in game-theoretic modeling for applications such as cooperation in wireless networks (Jaramillo and Srikant, 2010) and human computation systems (Ghosh, 2013). Assume that reputation is function of work ω for each contract which in turn is a function of the IPA. Thus, the higher the perception of an increased payoff, the higher is the probability that the task will be modeled as a contract and a two-player coalition will be formed to complete the task. Let $\omega_{1}$ and $\omega_{2}$ be the work performed by first mover and second mover respectively.

$$\omega =\omega_{1}+\omega_{2}$$ (6)

Assume $q_{1}$ and $q_{2}$ are number of coalitions that the first and second mover are a part of respectively. Thus, $q=q_{1}+q_{2}$ is the total number of coalitions. We assume an inverse resource function, where number of coalitions is inversely proportional to the work required for all coalitions. A detailed treatment of inverse resource functions in (Agaltsov et al., 2018). Thus, for some constant N, we have

q=N−ω, (7)

We now have the framework to obtain the collective payoff from a coalition. The collective payoff function $\left(p_{i}\right)$ is modeled by assuming that the more one player expends resources and works harder, the less valuable it is to the other player. Thus, the collective payoff function represents the utility (profits) of both players through the terms $p_{1}$ (player 1) and $p_{2}$ (player 2). To accomplish this, we use the Cournot duopoly model, a variant of which was first introduced by Cournot in 1838 (Tadelis, 2012). The Cournot duopoly model describes two identical players, players 1 and 2, producing some good. In our case, this good is the outcome of the work performed as a consequence of engaging in the contract. Assume that there are fixed costs of engaging in the work, and that the variable cost to each player i of performing the work is given by the cost function, $c_{i}=a\omega_{i}^{2}$ for i∈{1,2}. Demand for the work is given by q=N−ω, where $\omega =\omega_{1}+\omega_{2}$. Thus, for two players (player 1 and player 2) producing work $\omega_{1}$ and $\omega_{2}$ respectively, the payoff function $p_{i}$ is given by:

$$p_{i}=q\omega_{i}-a\omega_{i}^{2}$$ (8)

Here, i∈ {1, 2} for players 1 and 2 and a denotes variable costs. Thus, substituting Eq. 8 in Eqs. 6, 7, we get

$$p_{i}=\{\begin{array}{cc} \left(N-\omega_{1}-\omega_{2}\right)\omega_{1}-a\omega_{1}^{2} & \omega_{1}+\omega_{2}<N\\ -\alpha \omega_{i}^{2} & \omega_{1}+\omega_{2}\geq N \end{array}$$ (9)

We are implicitly assuming that total work ω cannot fall below zero, so that if both players together work to produce more than N, the work will be zero and a player’s payoff will be her costs.

For best response, we set

$$\frac{\text{d}p_{i}}{\text{d}\omega_{1}}=0$$ (10)

We get

$$\omega_{1}=\frac{N-\omega_{2}}{2\left(1+a\right)}$$ (11)

Since work ω is a function of initial payoff awareness, from Eq. 4, we have

$$\omega_{1}=f\left(\frac{\delta \lambda }{2}\right)$$ (12)

$$\omega_{2}=f\left(\frac{\delta \mu }{2}\right)$$ (13)

Without loss of generality, we assume constants $\delta_{1}$ and $\delta_{2}$ such that the above equations are expressed as

$$\omega_{1}=\frac{\delta_{1}\lambda }{2}$$ (14)

$$\omega_{2}=\frac{\delta_{2}\mu }{2}$$ (15)

For simplicity, the constants $\delta_{1}$ and $\delta_{2}$ are assumed to be linear functions of the constant δ which is the difference in perception of resource consumption for both players.

Substituting in Eq. 11, we get an equation for the relationship between the payoff factors at best response and obtain the ambiguity ratio (AR) of the first and second movers, λ/μ, as follows.

$$\text{Ambiguity}\;\text{Ratio}=\frac{\lambda }{\mu }=\frac{2N-\mu \delta_{2}}{2\mu \delta_{1}\left(1+a\right)}$$ (16)

Thus, λ∝−μ. This shows that the higher the difference in perception and actual values of ambiguity (λ) for the first mover, the lower is the difference in perception and actual values of ambiguity for the second mover (μ).

Substituting this value of λ in the equation for collective payoff, we observe the performance of the collective payoff at best response as follows

$$p_{coll_{i}}=\frac{{\left(2N-\mu \delta_{2}\right)}^{2}}{16\left(1+a\right)}$$ (17)

## Derivation of the Shapley Value for 2-Player Contracts

In this section, we analyze the two-player coalition to examine the contribution of each player to the coalition. To do this, we use the results from Eqs. 4, 5 from the above section. These results show the work of the first mover $\left(P_{1}\right)$ and second mover $\left(P_{2}\right)$, which is proportional to their IPA. Further assume that when the two players work together in a coalition, the players each can reduce their work load. These reductions are denoted as $\tau_{1}$ and $\tau_{2}$ for the first mover and second mover respectively. Thus, the contribution matrix of the individual players and the coalition are summarized in Table 3.

**TABLE 3:** Contribution matrix of coalition.

Player(s)	Contribution
$P_{1}$	$\frac{\delta \lambda }{2}$
$P_{2}$	$\frac{\delta \mu }{2}$
$P_{1},P_{2}$	$\left(\frac{\delta \lambda }{2}-\tau_{1}\right)+\left(\frac{\delta \mu }{2}-\tau_{2}\right)$

The Shapley value offers a way to quantify the contribution of each player to the coalition. In machine-learning applications, the Shapley value has been widely used to determine the value of a particular set of features to the overall representation of the dataset. For example, in seeking to determine a predictor for longevity, which feature contributes most to longevity from a pool of features including education, genetics, diet, and others. The seminal paper by Shapley (Shapley, 1953) describes how to determine the value of the expected marginal contribution of a coalition by considering all possible orders in which coalitions can be formed between players and assigning each player her marginal contribution.

Applying this technique to the two-player coalition, we get the marginal contribution matrix (Table 4). Specifically, the Shapley value emphasizes the role of timing. If the coalition is formed with $P_{1}$ first and then $P_{2}$ as shown in the first row, the marginal contributions of $P_{1}$ and $P_{2}$ are given as follows:

**TABLE 4:** Marginal contribution matrix.

Order of players	Marginal contribution $P_{1}$	Marginal contribution $P_{2}$
$P_{1}P_{2}$	$\frac{\delta \lambda }{2}$	$\frac{\delta \mu }{2}-\left(\tau_{1}+\tau_{2}\right)$
$P_{2}P_{1}$	$\frac{\delta \lambda }{2}-\left(\tau_{1}+\tau_{2}\right)$	$\frac{\delta \mu }{2}$

Averaging the columns, we get *P*_1_’s contribution to the coalition as shown in Table 4.

*P*_1_’s contribution to the coalition:

$$\frac{\delta \lambda -\left(\tau_{1}+\tau_{2}\right)}{2}$$ (18)

Similarly, from Table 4, we get *P*_2_’s contribution to the coalition as

$$\frac{\delta \mu -\left(\tau_{1}+\tau_{2}\right)}{2}.$$ (19)

Dividing Eq. 18 by Eq. 19, we obtain the marginal contribution ratio (MCR) of players $P_{1}$ with respect to $P_{2}$ as follows.

$$\text{Marginal}\;\text{contribution}\;\text{ratio}=MCR=\frac{\delta \lambda -\left(\tau_{1}+\tau_{2}\right)}{\delta \mu -\left(\tau_{1}+\tau_{2}\right)}$$ (20)

## Results

**FIGURE 3:**
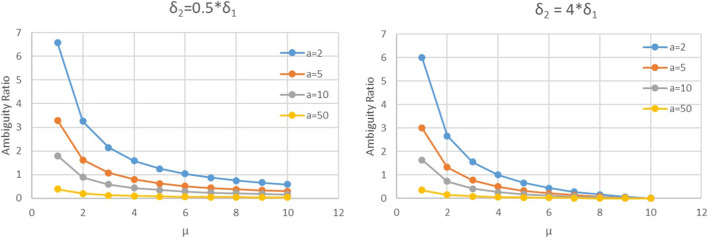
Relationship between the ambiguity ratio (AR) as a function of μ, for N=100 and $\delta_{1}=5$. As the variable costs (denoted by a) increase, the ambiguity ratio decreases. Further, as the difference between actual and perceived resource commitment levels of the second mover increase, the value of λ decreases.

Figure 3 shows the relationship between the ambiguity ratio (AR) and the difference in perceived and actual ambiguity levels for the second mover (μ) at best response. From sub-Figures 3, we see the inverse relationship between AR and μ. Further, as the variable costs (a) increase, AR decreases signifying an inversely proportional relationship between the AR and a. Additionally, AR is dependent on the difference in perceived and actual resource commitments of the first mover $\left(\delta_{1}\right)$ and the second mover $\left(\delta_{2}\right)$. From Figure 3, we see that as the ratio $\delta_{2}/\delta_{1}$ increases from 0.5 to 4, it signifies either an increase in the ambiguity perception difference of player 2 (μ) or a decrease in the ambiguity perception difference of player 1 (λ). This increase in the value of the ratio $\delta_{2}/\delta_{1}$ implies that since λ is inversely proportional to μ, the ambiguity ratio (AR) is inversely proportional to the ratio given by $\delta_{2}/\delta_{1}$.

The findings in Figure 3 follow from Eq. 16, which show AR and the variable costs a are inversely proportional. This relationship is due to the additive inverse relationship between the work performed by player 1 $\left(\omega_{1}\right)$ and the costs (a), as seen in Eq. 11. Since $\omega_{1}$ is directly proportional to the difference in ambiguity perception in player 1 (λ), an increase in $\omega_{1}$ signals an increase in the AR as well. This confirms that an increase in the costs a is related to lower amount of work performed by player 1 $\left(\omega_{1}\right)$. Since work performed is a function of the AR, as a increases, the AR decreases.

**FIGURE 4:**
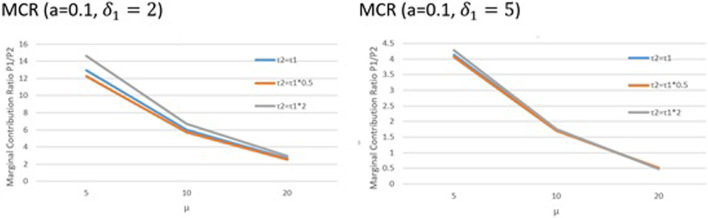
Shapley value findings. The variation of the marginal contribution ratio of the two players is explored as a function of the difference in perceived and actual ambiguity levels for the second mover (μ), variable costs (a), and the difference in perceived and actual resource commitments of the first mover $\left(\delta_{1}\right)$.

Next, we present the findings of the Shapley values of players involved in the coalition in terms of the marginal contribution ratio (MCR). The MCR findings depicted in Figure 4 are derived from Eq. 20. From Figure 4, we see that as μ increases, MCR decreases. The MCR is directly proportional to the AR, and affirms the inversely proportional relationship between the ambiguities experienced by the two players (λ,μ) that was shown in Figure 3. This continues in the form of decreased contribution by each player to the coalition resulting in a decrease in the MCR. As $\tau_{2}/\tau_{1}$ increases, MCR increases. The variables $\tau_{1}$ and $\tau_{2}$ denote the benefits to the first mover $P_{1}$ and second mover $P_{2}$ respectively in the form of reduction in work loads due to the formation of a coalition. We see that an increase in $\tau_{2}/\tau_{1}$ denotes an increase in the benefits to each player from the coalition and so each player brings higher value to the coalition which is manifested in the form of an increase in the MCR. As $\delta_{1}$ increases, MCR decreases. An increase in the resource commitment decreases the MCR for the players since it denotes tasks of increasing complexity. These findings strengthen timing in contractual relationships. The order in which nodes commit to the contract matters. The first-mover and second-mover advantages vary, and the ambiguity of the contract impacts the marginal contributions of each node in the coalition.

## Kernel Representation for Contractual Graphs

In this section, we propose a simple kernel for contractual graphs that depict marketplaces of two-player coalitions formed to complete ambiguous tasks. Specifically, we address the question of how we can determine if two marketplaces of ambiguous tasks are similar. The implications of this question are several that range from evaluating marketplaces with similar resources, players and tasks. Several metrics can be used to study the effectiveness of a marketplace, such as number of coalitions, average size of coalitions and average task completion rate. An analysis of kernels of successful marketplaces can yield insights about the factors that significantly impact the efficiency of a marketplace of ambiguous tasks.

Since our model assumes two-player coalitions, we use the Shapley value of a two-player coalition developed in the previous section to build the kernel. Assume there are n players in a marketplace, which results in potential n!/(n−2)! coalitions of two-players. A player can be a part of multiple coalitions. Each coalition represents an edge on the contractual graph, and the weight of the edge is denoted by the Shapley value of the coalition. As derived in the above section, the Shapley value is dependent on the order in which the players form the coalition.

**FIGURE 5:**
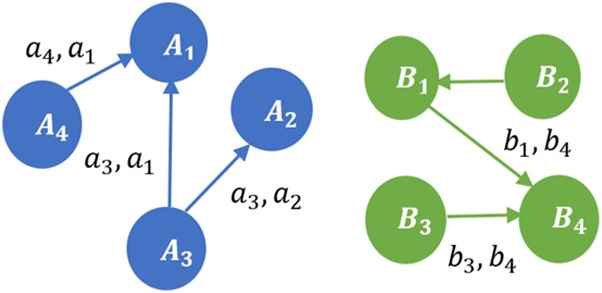
Contractual graphs $G_{1}$ **(left, in blue)** and $G_{2}$ **(right, in green)** are depicted. Each directional edge represents a contract in a 2-player coalition to complete an ambiguous task. The Shapley value of a coalition is the weight of each edge. For example, the edge with weight $\left(a_{4},a_{1}\right)$ represents the coalition $A_{4}A_{1}$.

Consider two contractual graphs in Figure 5. The Shapley value of each coalition is marked on the edge. Two graphs are considered similar if the sums of Shapley values of coalitions are equal.

We now present the conditions under which two contractual graphs are considered similar. Let graph $G_{1}\equiv \left(V_{1},E_{1}\right)$, where $V_{1}$ is the finite set of vertices in graph $G_{1}$. Let $E_{1}$ be the set of edges in graph $G_{1}$, such that $E_{1}\subseteq \left\{\left\{u,v\right\}\subseteq V_{1}|u\neq v\right\}$. An edge (u,v) denotes a coalition in which u is the first mover and v is the second mover in the coalition. Assume that there are N tasks of varying ambiguity in a marketplace. Consider another contractual graph $G_{2}$, such that $G_{2}\equiv \left(V_{2},E_{2}\right)$. Similar to $G_{1},E_{2}$ and $V_{2}$ are the set of vertices and edges in $G_{2}$ respectively. Let the number of edges in graphs $G_{1}$ and $G_{2}$ be denoted by $n_{1}$ and $n_{2}$ respectively.

Two graphs $G_{1}$ and $G_{2}$ are similar if and only if they satisfy the following three properties.

1. Edge cardinality equivalence: The number of edges in graph $G_{1}$ is equal to the number of edges in graph $G_{2}$, i.e., $n_{1}=n_{2}=n$.
2. First mover equivalence: The sum of the Shapley values of the first movers in graph $G_{1}$ is equal to the sum of the Shapley values of the first movers in graph $G_{2}$. Thus,

$$\sum_{i=1}^{n}u_{i-G_{1}}=\sum_{i=1}^{n}u_{i-G_{2}}$$ (21)

where, $u_{i-G_{1}}$ and $u_{i-G_{2}}$ are the Shapley values of first movers in graphs $G_{1}$ and $G_{2}$ respectively.

3. Second mover equivalence: The sum of the Shapley values of the second movers in graph $G_{1}$ is equal to the sum of the Shapley values of the second movers in graph $G_{2}$. Thus,

$$\sum_{i=1}^{n}v_{i-G_{1}}=\sum_{i=1}^{n}v_{i-G_{2}}$$ (22)

where, $v_{i-G_{1}}$ and $v_{i-G_{2}}$ are the Shapley values of second movers in graphs $G_{1}$ and $G_{2}$ respectively.

These properties can be generalized to k contractual graphs to establish an upper bound on the Shapley values of all coalitions.

### Generalized Expression for Similarity of Contractual Graphs

The upper bound of the sum of the Shapley values of the coalitions in k contractual graphs is shown below, where the right-hand side of the expression denotes the upper bound as given by

$$y\leq \sum_{s=1}^{k}\left(\sum_{i=1}^{n}\left(u_{i-G_{s}}+v_{i-G_{s}}\right)\right)$$ (23)

The upper bound in Eq. 23 states that for a given contractual graph, the sum of the Shapley values of the first-movers and the second movers for all coalitions in that graph represents the term $\left(u_{i-G_{s}}+v_{i-G_{s}}\right)$, where i denotes the number of coalitions in a graph s. Taking the summation of this over s=1,2,…,k graphs, we obtain the upper bound on the Shapley value of coalitions in k graphs denoted by $\sum_{s=1}^{k}\left(\sum_{i=1}^{n}\left(u_{i-G_{s}}+v_{i-G_{s}}\right)\right)$. The upper bound signifies that any modification to the structure of a contractual graph, either by adding players to a coalition, or deleting players will alter the structure of the contractual graph. Consequently, this will result in a new contractual coalition with different Shapley values. Thus, the upper bound is sensitive to the contractual graph structure, and offers a metric with which to gauge coalition formation in contractual graphs. A metric defined by this upper bound may serve several applications such as in limiting the number of coalitions in transactional environments, regulating abnormal disparities between first-movers and second-movers, investigating the viability of underperforming marketplaces, and in studying the factors that contribute thriving marketplaces.

## Future Work

This paper presented an introduction to the theory of contractual graphs, by modeling contracts are commitments between two players to complete an ambiguous task. The theory of contractual graphs can be extended in several ways, some of which are summarized below.

### Role of Incentives

While ambiguity of tasks can represent hurdles in task completion, incentives might be able to alleviate some of the costs involved in accepting tasks with high ambiguity. Additionally, incentives might be able to nudge the formation of coalitions thereby encouraging second movers to collaborate frequently with first movers on task completion. The role of incentives in economic literature has been widely studied (Bénabou and Tirole, 2006; DeMarzo and Saninkov, 2016). In (Bénabou and Tirole, 2006), where the authors explore the basis of incentives in psychology. Specifically, the role of incentives in intrinsic, extrinsic and reputational motivations have been considered in the development of game-theoretic models to gain insights into individual contributions and interactions. Graph theoretic representation learning can be enriched with the incorporation of additional sociological constructs such as identity (Akerlof and Kranton, 2000) to explain outcomes of interactions between nodes.

### Negotiations Over Time

Our model for studying the performance of two-player coalitions in solving ambiguous tasks uses a single-stage contract. That is, the first mover assesses the ambiguity and creates a contract that the second mover accepts. In practice, however, contract formation goes through multiple stages of bargaining and negotiations over the terms of the contract. Additionally, problems related to imperfect information such as moral hazards and adverse selection can significantly impact the outcome of the contract formation, interpretation and execution.

### Spectrum of Perceived Ambiguity

In our model, we chose a binary system for modeling ambiguity (high/low). While easier to model binary choices, in practice, ambiguity lies along a spectrum. Understanding the impact of ambiguity for a range of values between 0 and 1 can help further illustrate its impact on the formation of a contract.

### Backing off From Contracts with Penalties/Prorated Benefits

While our model assumes that players commit to the contract and stay committed until task completion, it would be worth investigating how players would behave if they had the option to exit the contract. Similar to real-world situations where premature contract termination results in penalties or prorated rewards, modeling a marketplace of ambiguous tasks with players who have the option of exiting the contract would provide insights into real-world situations. Examples of such situations include students who sign up for classes and withdraw, renters who terminate the lease before its due date and employees who leave prior to the end of their probationary period. In all of these cases, embedded penalties/prorated rewards exist in the contractual terms and it would be worth investigating those through the lens of ambiguity. The players who have exited the contract are now freelancers of some sort, and how they impact the dynamics of the marketplace would be an interesting direction for future research.

### Coalitions of Multiple Players

While our model chose two-player coalitions as dyadic exchanges, extending this model to n-player coalitions would be beneficial. Our model also does not account for non-dyadic exchanges involving a single player, i.e., n=1. These scenarios are indicative of transactions that represent self-loops in graphs (n=1), or longer chains (n>2). Understanding how such coalitions of n, where n≠2 players react to ambiguous tasks and each other’s perceptions of ambiguity will have a significant impact on the performance of the coalition.

## Conclusion

Contractual graphs arise in multiple situations. The agents involved in contractual graphs form connections among themselves upon the fulfilment of underlying conditions. For example, sending a packet from one node to another only when the packet is received with minimal distortion can be viewed as contract between two nodes only to transmit high-fidelity data. Another example of a contractual graph could be the formation of a connection in a social network, where two individuals form a “friend” connection only if they are separated by n friends. In this case, the contract is fulfilled only if there exist fewer than n degrees of separation. Contractual graphs can be used to study a variety of graphs where the formation of edges is dependent upon the completion of specific conditions. However, the conditions underlying the formation of an edge are not always specific enough to be readily automated in software. This motivates the need for a study into how ambiguity impacts contract formation. Thus, we studied ambiguous marketplaces, defined as marketplaces containing tasks of varying ambiguity that players could seek to complete in two-player coalitions.

Our findings showed how the perception of ambiguity in the contract impacts the resources allocated by a player to the task, thus displaying ambiguity aversion. Nodes model the contract by observing the parameters of the contract and allocating resources for completing the tasks specified in the contract. Using a game-theoretic formulation, we developed a model that assigns payoffs to players for the tasks completed. We distinguished between how the order in which players proceed to enter the two-player coalition affects the value that each player brings to the coalition. This metric was quantified using the Shapley value, that showed how the ambiguity of the task, costs of performing the task and resource allocation all played a part in determining the performance of the coalition. The Shapley value of a two-player coalition was then used to assign a tuple of edge weights. The set of all edge weights then formed a graph kernel for the contractual graph. We derived the conditions under which two contractual graphs are similar using edge cardinality, first-mover and second-mover equivalence. Finally, we proposed an upper limit on the sum of Shapley values of coalitions in k contractual graphs.

Contractual graphs offer several avenues for exploration due to their importance to network science. For example, we have assumed two-player coalitions where players complete the task, once they enter into the contract. In practice, however, coalition sizes may vary and players may have the option to leave the coalition even after they have entered into a contract. Similarly, new players may join the coalition even after the task has begun execution. Contractual graphs can be studied broadly, as well as in several niche environments. A better understanding of the behavior of contractual graphs can lead to the development of effective representative kernels, which in turn would facilitate learning about graphs.

## Data Availability

The raw data supporting the conclusion of this article will be made available by the author, without undue reservation.
